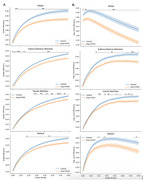# Cognitive impairment, brain connectivity, and long‐Covid: understanding the complex interplay

**DOI:** 10.1002/alz.091565

**Published:** 2025-01-09

**Authors:** Micaela A Hernández, Fermin Travi, Juan Kamienkowski, Bruno Bianchi, Agostina Carello, Belén Helou, Lucia Crivelli, Diego Fernández Slezak, Gustavo Sevlever, Ricardo Allegri, Ismael Luis Calandri

**Affiliations:** ^1^ Fleni, Buenos Aires, Buenos Aires Argentina; ^2^ Facultad de Ciencias Exactas y Naturales (FCEyN), Buenos Aires, Buenos Aires Argentina

## Abstract

**Background:**

The persisting cognitive symptoms observed in individuals after a Covid‐19 infection (long‐Covid) remain an enigmatic aspect of the disease. Clinical descriptions in the literature are dissimilar, suggesting more than one clinical phenotype. Our study aims to explore cognitive performance and brain network of long‐Covid patients, to determine the neural correlates of cognitive impairment.

**Method:**

Case‐Control study of individuals with cognitive complaints persisting at least 12 weeks after Covid‐19 infection, and age‐ and sex‐ matched healthy controls without history of covid‐19 infection. We performed a brain MRI with rs‐fMRI sequences and a neurocognitive test (NCT) based on the UDS‐3 protocol. We generated z‐scored neuropsychological data, normalized to the normal population, and averaged them to create 7 cognitive domains. We segmented rs‐fMRI data into seven distinct functional neural networks (Salience/Ventral Attention, Dorsal Attention, Default, Frontoparietal, Visual, Somatomotor, and Limbic) and measured network efficiency, largest connected component, average participation coefficient, and modularity. We applied a Wilcoxon test to assess the significance of differences in measurements over networks.

**Result:**

Forty‐two subjects were recruited, 30 were female. Mean age 56 (±12). The control group consisted of 43 subjects, 25 were female. Mean age 59 (±9). The NCTs scores yielded statistical significant differences in long‐Covid subjects compared to controls, at memory (mean difference:‐1.12, IQR: ‐1.78; ‐0.32), attention (mean difference: ‐1.21, IQR: ‐1.80; ‐0.40), executive (mean difference: ‐0.82, IQR: ‐1.37; ‐0.12), and global composites (mean difference: ‐0.87, IQR: ‐1.36; ‐0.25). Regarding functional networks, we observed significant differences (p < 0.001) in the global and mean local efficiency of the Salience/Ventral Attention and Global networks, and to a lesser extent (p < 0.005 and p < 0.01) in the Default and Dorsal Attention networks (Figure 1).

**Conclusion:**

Our study identified that long‐Covid subjects differ in their cognitive performance compared to controls with less effective and organized connection among Salience/Ventral Attention and Global networks.